# Effects of Translucency and Thickness of Lithium Disilicate-Reinforced Glass-Ceramic Veneers on the Degree of Conversion of a Purely Light-Curing Bonding Resin: An In Vitro Study

**DOI:** 10.3390/polym15071617

**Published:** 2023-03-24

**Authors:** Anthony Poca, Kenza De Peretti Della Rocca, Karim Nasr, Romain Ducassé, Thibault Canceill

**Affiliations:** 1 Département Odontologie, Faculté de Santé, Université Paul Sabatier, Hôpitaux de Toulouse, 3 Chemin des Maraîchers, CEDEX 9, 31062 Toulouse, France; 2 French Institute of Metabolic and Cardiovascular Diseases (i2MC), InCOMM (Intestine ClinicOmics Microbiota & Metabolism) UMR1297 Inserm/Université Toulouse III, 31432 Toulouse, France

**Keywords:** light-curing composite resin, dental ceramics, glass-ceramics, FTIR spectroscopy

## Abstract

The objective of this study was to evaluate the variations in the degree of conversion (DC) of a light-curing composite resin when the thickness or the translucency of lithium disilicate-enriched glass-ceramic veneers are modified. IPS e. max^®^ CAD blocks of the MT-A2, LT-A2 and MO1 types were cut to obtain four slices with thicknesses ranging from 0.6 mm to 1 mm. A strictly light-curing composite resin (G-aenial Universal Injectable) was injected in the empty part of a silicone mold so that the veneer could then be inserted under digital pressure to the stop. A 40 s light cure (1400 mW/cm^2^) was then performed. Resin samples were analyzed using Fourier transform infrared (FTIR) spectroscopy. When the degree of translucency of the ceramic was modified, a decrease in the resin conversion rate was noted, but with a non-significant global *p*-value (*p* = 0.062). Interestingly, the degree of conversion of the light-curing composite resin was also modified when the ceramic’s thickness increased, especially when it was over 1 mm (DC_0.6_ > DC_0.7_ > DC_0.8_ > DC_1_; *p* < 0.0001). This confirms that the degree of conversion of a bonding material is very dependent on the ceramic’s thickness. Contradictory data are, however, found in the literature, where there are reports of an absence of a difference between the DC obtained with thicknesses of ceramics of 0.7 and 2 mm.

## 1. Introduction

The use of ceramics is constantly increasing in dentistry, and they could be even more used if the use of alloys is gradually reduced in the future due to the evolution of legislation [1]. Dental ceramics are a family of materials bringing together glass-ceramics (reinforced or not) and polycrystalline ceramics. Their optical, mechanical and physical properties are often close to those of natural dental tissues. These materials are described as having many possibilities with regard to translucency and brightness, a wide range of shades and an excellent biocompatibility [2,3]. Feldspathic ceramics and other glass-ceramics contain less fillers in their matrices, and thus they present a lower mechanical resistance than crystalline ceramics, which are also known as zirconia [4,5]. However, the vitreous matrix allows the structures which contain it to have more interesting optical properties and to be more aesthetic. The best compromise in terms of aesthetics and strength for all-ceramic monolithic restorations is lithium disilicate-enriched glass-ceramic [6]. Its suitability in numerous indications, as well as its different packaging, makes it the most commonly used ceramic in dentistry [3]. In recent years, the development of digital technology has made machining an increasingly common process not only in prosthetic labs, but also in practice, since dental practitioners may now invest in their own machining line. This technique, which is faster than the hot-pressing one, also guarantees precision as it appears to be at least as accurate as more traditional techniques [7,8], reducing operator-dependent errors. 

In daily dental practice, it is common to use several thicknesses of ceramics depending on the treatment applied on a tooth. Due to the dynamic of minimally invasive dentistry and thanks to the ceramics’ properties, the minimum thickness can range from a few tenths of a millimeter for pellicular veneers to more than 2 mm for some onlays or overlays. Veneers are defined as thin ceramic restorations that are bonded to dental tissues, are especially designed for anterior teeth and can take different forms depending on the preparation chosen: for example, pellicular (or fenestrated) veneers with a reduction in the free edge without palatal return (goal margin), or even with incisal overlap. Although they can be used to partially hide the dental substrate if it shows a shade anomaly [9], veneers cannot be applied on very discolored teeth because of the translucency of the ceramic. This parameter is a key optical biomimetic element for replicating the visual appearance of a natural tooth. It depends on the way a material allows light rays to pass through it and transfer inside its structure [10]. Translucency is the opposite of opacity, which is the capacity of a medium to absorb and reflect a light ray that cannot pass through it and is therefore not transmitted.

The method of choice for assembling ceramic veneers onto dental tissues is bonding through composite resins [2]. Indeed, the low retentivity of the surfaces contraindicates cementation, and moreover, an increased longevity of the ceramic is observed when bonding is performed [11]. Among the composite resins that do not contain a proper bonding potential are found purely light-curing materials and dual-curing materials that present a photopolymerizable part and a chemopolymerizable part. The polymerization reaction of a composite resin follows a chain addition of monomers present in the matrix under the action of an activator, which will change according to the type of resin used. The polymerization mechanism of the purely light-curing material is based solely on the irradiance [12,13], and thus, it should be reduced as little as possible. That is why this type of bonding resin may only be used in favorable conditions in terms of ceramic thickness, shade and translucency [3,14,15,16,17]. However, it is increasingly used in dentistry because of its other advantages: the absence of a tertiary amine in its composition gives it a better chromatic stability, its working time is almost infinite and the management of excess is easier [14]. In contrast to dual-curing materials, the polymerization is not exothermic and cannot lead to pulp toxicity and post-operative sensitivities [11,17]. Indeed, the material’s temperature can vary during its polymerization depending on its structure, the time of irradiation and the mode selected on the lamp [18,19]. As there is no true consensus on the temperature above which the risk of pulp necrosis is significantly aggravated [19], it is recommended to choose the continuous irradiation mode to light cure a composite resin [18]. 

The challenge of ensuring correct polymerization of the luting material is allowing the ceramic restoration to have a strength and durability compatible with the patient’s function [11]. In addition, a poor polymerization rate can lead to toxicity due to residual monomers, a higher solubility and a chromatic instability [16,17,20]. The degree of conversion, or polymerization rate, indeed corresponds to the proportion of monomers that have been linked together during the chain reaction. Even if it is not, by itself, the guarantee of an efficient polymerization, it is the most representative characteristic of the process [20]. A clinically acceptable degree of conversion is in the range of 60 to 75% [2,20], as it will ensure sufficient physical, mechanical and optical properties. Similarly to the temperature increase in the material during the polymerization, its conversion rate depends on the time and the mode selected on the lamp [21]. The option of continuous irradiation with respect to the time indicated by the resin manufacturer remains the best recommendation. In addition, for the last ten years, an additional issue has emerged in the challenge of polymerization. The questions related to the safety of use of composite resins, whether they are indicated for direct restoration or for the bonding of prostheses, regarding the possible release of monomers in the organism has led to the need to optimize as much as possible the conversion rate of these materials. 

Thus, the objective of this study is to evaluate the variations in the degree of conversion of a light-curing composite resin when the thickness or the translucency of lithium disilicate-enriched glass-ceramic veneers is modified. The null hypothesis is that the polymerization rate of the bonding material does not vary when the ceramic thickness or its translucency are changed.

## 2. Materials and Methods

An in vitro study was carried out in 2022 in the Odontology Department of the Toulouse Health Faculty (Université Toulouse III, Toulouse, France). All the biomaterials used in this study are presented in Table 1.

### 2.1. Veneer Preparation

Pre-crystallized IPS e. max^®^ CAD blocks of the MT-A2, LT-A2 and MO1 types (Ivoclar Vivadent, Schaan, Liechtenstein) were cut with a low-speed diamond blade under irrigation (IsoMet Low Speed Saw^®^, Buehler, Lake Bluff, IL, USA) to obtain four slices with thicknesses ranging from 0.6 mm to 1 mm (0.6, 0.7, 0.8, 1 mm) for the MT-A2 block and one 1 mm thick slice from the LT-A2 and MO1 blocks. These samples of calibrated thicknesses were considered as the veneers for this study. They were then fired in a Programat CS^®^ furnace (Ivoclar Vivadent, Schaan, Liechtenstein) according to the program indicated for each degree of opacity: “Cristall/Glaze” for MT-A2 and LT-A2 and “Impulse” for MO1. The six veneers were polished on one side using a polishing burr dedicated to ultra-fine-grained ceramics (Ref. No. 952.040HP, Stoner, Toulouse, France) and fixed on a handpiece without irrigation. Their thicknesses were all checked at the end of polishing with a 0.1 mm precision digital caliper.

### 2.2. Translucency Parameter

The determination of translucency was performed by calculating the veneers’ Translucency Parameter (TP) (n = 10 in each group). The L*a*b* colorimetric data were measured 20 times for each veneer by using a UV spectrophotometer (EFI ES-1000 i1, X-rite, MI, USA) connected to the EFI Color Profiler^®^ software (X-rite, Grand Rapids, MI, USA). These recordings were carried out successively on ideal black (10 times) and white (10 times) backgrounds of a calibrated gray scale (TrueColors, Scuadra, France). The spectrophotometer was calibrated before each measurement. Equation (1) was finally applied to calculate the TP score.

TP = [(L*_white_ − L*_black_)^2^ + (a*_white_ − a*_black_)^2^ + (b*_white_ − b*_black_)^2^]^1/2^ (1)

where L*_white_, as an example, corresponds to the mean obtained from the 10 L* values on a white background for each sample.

### 2.3. Bonding Protocol

A specifically designed mold made of silicon (Aquasil Soft Putty Regular Set, Dentsply Sirona, Bensheim, Germany) and polytetrafluoroethylene, positioned on a glass board, allowed us to reproducibly set all the veneers by leaving a 500 µm space for the future composite resin. The choice of a resin thickness of less than 1 mm allowed us to eliminate any risk of differential polymerization when interpreting the results. 

At first, all the samples were inserted on the mold with the polished side up to validate their correct repositioning. They were then cleaned with a 70% alcohol solution before proceeding to the bonding protocol. In this study, the sample manipulation was shared between two calibrated and trained operators. The experiments were performed in a room with low lighting and a temperature of 25 degrees Celsius. A strictly light-curing composite resin (G-aenial Universal Injectable, A2 shade, GC Dental, Tokyo, Japan) was injected in the empty part of the mold so that the veneer could then be inserted under digital pressure to the stop. A 40 s light cure (1400 mW/cm^2^, D-Light Pro, GC Dental, Tokyo, Japan) was finally performed by positioning the lamp tip to be in close contact with the polished surface of the lithium disilicate-enriched veneer (dimensions 12.4 × 14.5 mm).

Resin samples, as they were not chemically linked to the ceramic nor to the glass board, could be delicately separated from their support with a scalpel blade. One uncured control specimen was also created on the day of the infrared analyses. 

### 2.4. Infrared Spectroscopy

The degree of conversion of the composite resin was analyzed seven days after the light-curing protocol to take into account the post-polymerization that could have occurred after the experiments. In total, 0.005 g of each sample was isolated, manually crushed and mixed with 0.4 g of potassium bromide (KBr). The degree of conversion among the specimens was calculated using Fourier transform infrared (FTIR) spectroscopy (Spectrum One, Perkin Elmer, MA, USA) configured via an absorbance mode for measurements between wavelengths of 400 cm^−1^ and 4000 cm^−1^ (n = 10 for each group of translucency and n = 8 for each group of thickness). Background spectra were launched prior to running samples through an empty mold. The pellets were then placed inside the FTIR chamber and cured spectra were collected. The conversion degree was calculated as the change concerning the ratios between the intensities of the absorbance peaks corresponding to the aliphatic (1638 cm^−1^) and aromatic (1608 cm^−1^) C=C bonds, which was always in relation to the unpolymerized material [17]. Precisely, the following equation (Equation (2)) was applied to calculate the degree of conversion (DC%) [22]:

(2) $$DC\%=\left(1-\frac{\left(\frac{1638\;{cm}^{-1}}{1608\;{cm}^{-1}}\right)cured}{\left(\frac{1638\;{cm}^{-1}}{1608\;{cm}^{-1}}\right)uncured}\right)\times 100$$

### 2.5. Statistical Analyses

Results for TP and DC% all consist of quantitative variables. They are thus presented as mean ± standard deviation. Comparisons between the groups (of thickness and translucency) were performed with ANOVA tests after the double verification of the normal distribution for the values and the equality of the variances. Tukey post hoc tests were then applied to compare groups by pairs. All the tests were launched on Stata v.13.0 (StataCorp, College Station, TX, USA) and Prism 5 (GraphPad, San Diego, CA, USA). The level of significance was set at a *p*-value ≤ 0.05.

## 3. Results

### 3.1. Translucency Parameter

As expected, mean values for the Translucency Parameter increase as the degree of opacity of the ceramic decreases (Table 2). The differences are particularly significant between the groups MO1 vs. MT A2 and LT A2 vs. MT A2.

### 3.2. Influence of Ceramic’s Translucency

Even if the degree of conversion tends to decrease when the degree of translucency of the ceramic increases (DC_MO1_ < DC_LT-A2_ < DC_MT-A2_), the global *p*-value is not statistically significant (*p* = 0.062) (Table 3). When compared in pairs, the DC do not differ significantly either. The MO1 group presents a lower mean value for its DC than the others, but an important dispersion of values makes it difficult to draw conclusions about this group. Thus, the translucency of lithium disilicate-reinforced ceramics does not appear to affect the degree of conversion of the purely light-curing composite resin. 

### 3.3. Influence of Veneer Thickness

Increasing the thickness of the ceramic veneer significantly modifies the degree of conversion of the light-curing composite resin (DC_0.6_ > DC_0.7_ > DC_0.8_ > DC_1_; *p* < 0.0001) (Table 4). Precisely, the differences remain as non-significant between the three thinnest groups of the study, which all are significantly different from the 1 mm thick group.

## 4. Discussion

The use of a light-curing composite resin for bonding lithium disilicate-reinforced ceramic veneers presents many advantages [14], but the possible modification of the resin’s degree of conversion, as it may alter the quality of the bonding [20], had to be studied with variations in the ceramic’s translucency and thickness. The results of our study show that the opacity of the glass-ceramic veneer has no influence on the composite resin’s DC. However, the veneer’s thickness has a negative influence on the DC, especially at a thickness of 1 mm. The null hypothesis which stated that the light-curing composite resin’s DC would not vary with changes in the translucency or the thickness of the ceramic is thus partially rejected. Nevertheless, despite the tendency for the degree of conversion to decrease as the ceramic’s opacity increases, the results are not statistically significant. Similarly, although a trend is observed across all groups made with various thicknesses, only the values for the 1 mm thick veneers are significantly lower than those for the thinner ceramics.

Our results are in agreement with those of Martins et al., who concluded in their meta-analysis that the bonding material’s degree of conversion tends to be higher when the ceramic is thinner [3]. They also specified that a thickness of more than 1 mm consistently leads to a reduction in the DC, regardless of the resin-curing mode. The conclusions of the work of Runnacles et al. are also along the same lines, stating that the materials’ DC has a negative correlation with the ceramic’s thickness [14]. However, their results are only statistically significant for thicknesses above 1.5 mm. The trend is also similar in two other studies that showed a decrease in the degree of conversion for light-curing composite resins as a function of ceramic thickness [13,16].

Contradictory data are, however, found in the literature, with conclusions on the absence of a difference between the DCs obtained with thicknesses of ceramics of 0.7 and 2 mm [23]. These contrasting results may be explained by the use of a different bonding agent which was in this case a dual-curing composite resin. Our choice was to use a purely light-curing biomaterial because these materials are increasingly being used by dental practitioners for the bonding of veneers, even though this indication is not mentioned in the product datasheet. As Leung et al. recently pointed out in their review on luting materials in dentistry, the use of a purely light-curing resin is justified in the literature and by the practitioners because it is the most esthetic bonding material [24]. One of the main reasons why the material is not sold for bonding is its filler content, which is higher than that of resin cements and not optimized for thin-film spreading.

In our protocol, we have tried to reproduce a clinical situation as much as possible by polishing the external surface of the veneers. However, it was not possible to treat the surface in contact with the composite resin with hydrofluoric acid and silane, as the two materials needed to be easily separated. These two steps would also slightly modify the light penetration through the veneer [12,13], and consequently, modify the DC. In the study, we focused on one type of ceramic in particular, and we chose the MO-1, LT-A2 and MT-A2 opacities and shades because they correspond to the standard for anterior veneers. We have also considered the 1 mm thickness as the maximum thickness in our study, as it was recommended by Pascal Magne and Urs Belser in 2002 [25]. Usually, clinically bonded veneers range from about 0.3 to 0.8 mm, but the cutting equipment available in our lab did not allow us to slice at such a thin thickness. In the literature, it is possible to find significant results concerning the decrease in the resin’s DC whatever the ceramic used with various thicknesses [15], but there are also non-significant results. For example, Cho et al. found no difference when calculating the degree of conversion under veneers of thicknesses between 0.3 and 1.2 mm [11]. In this case, the polymerization time in their study can also explain, at least partially, the absence of a clear conclusion. The literature is also discordant regarding the results obtained with various translucencies. The work of Alghaith [26] shows results on the degree of opacity that are in agreement with our present study, emphasizing that the thickness factor is the only one with a statistically significant influence on the DC. However, these results are different from those of Calgaro et al. [27], who concluded that the degree of translucency was the most important factor influencing the degree of conversion. In any case, our data showing that the Translucency Parameters increase when the degree of opacity of the ceramic is lower are consistent with those of a previously published study using the same thickness settings [15].

Finally, our results and those found in the international literature allow us to affirm that the G-aenial Universal Injectable^®^ composite can be used in the assembly of lithium disilicate-enriched glass-ceramic veneers up to a thickness of 1 mm, without any clinical impact on its degree of conversion. Moreover, the DC obtained in the study is consistent with the values that allow for a clinically acceptable performance [3,20,28,29].

The conclusions can vary if the choice of the bonding material is orientated on another composite resin. For example, with the Variolink II^®^ luting composite (Ivoclar Vivadent, Liechtenstein), the DC obtained in the LT-A2 group appears to be statistically higher than that for the MO-A2 group, and is not significantly decreased compared to the control group without ceramic interposition [28]. It is also proven in several studies that the degree of conversion of light-curing bonding composites is significantly higher than for dual-curing biomaterials [3,11,16,27].

The quality of the polymerization of the resin influences its resistance, but also its stability in time. Thermogravimetric studies that force the separation of bonded species in a material show that, for purely light-cured composites, their stability can be more precarious when the recommended light-curing time has been halved [30]. However, there are important differences depending on the type of monomer used in the matrix. In our study, the material’s matrix is composed of Bis-GMA. Biomaterials of this type show the greatest tolerance to curing variations and wet storage [30].

Having validated the quality of the polymerization of the material under a lithium disilicate-enriched glass-ceramic veneer, it would now be interesting to investigate the fracture resistance of the ceramic-resin assembly. Currently, the studies conducted on this topic mainly evaluate biomaterials presented as luting cements, and therefore have a dual-curing mode. Their results generally confirm that the application and polymerization of a composite resin on a veneer increases its flexural strength [31,32].

However, all these results cannot replace the development of clinical studies to evaluate the behavior of dental veneers and their luting cement in clinical practice. In vitro procedures typically use ideal conditions, such as flat surfaces, that do not fully represent the complex and varied clinical situations encountered in real patients. Bonding techniques are also simplified and may not reflect the full range of clinical protocols that dentists may use [33]. It is highly complicated to reproduce the long-term performance of restorations in the oral environment, including in the presence of multiple biological factors such as saliva, blood and other oral fluids that can affect the bonding process. Therefore, in vitro studies can provide valuable insights concerning the properties and behavior of biomaterials, such as light-curing cements studied in this work.

## 5. Conclusions

The degree of conversion of a purely light-curing composite resin remains statistically the same when the opacity of a lithium disilicate-enriched glass-ceramic veneer increases. However, the DC decreases with increasing ceramic thickness. Despite a trend that could be observed in all groups, only the values for the 1 mm thick veneers appear to be significantly lower than those for the thinner ceramics. Under the same conditions, and for thicknesses greater than 1 mm, the literature seems to confirm that the use of a dual-curing material can be recommended in order to guarantee sufficient bonding quality.

## Figures and Tables

**Table 1:** Presentation of the biomaterials used in the study.

Biomaterials	Manufacturer	Chemical Components
IPS e.max CAD blocks	Ivoclar Vivadent, Schaan, Liechtenstein	Lithium disilicate glass-ceramic (SiO_2_, Li_2_O, K_2_O, P_2_O_5_, ZrO_2_, ZnO, Al_2_O_3_, MgO)
G-aenial Universal Injectable (A2 shade)	GC Dental, Tokyo, Japan	Light-curing flowable composite resin (CAS: 41637-38-1, 43048-08-4, 1985-51-9, 128-37-0, 9003-08-1, 75980-60-8, 72869-86-4, 2440-22-4, 109-16-0, 1879-09-0)

**Table 2:** Mean values (±SD) for the Translucency Parameter in the three groups concerning 1 mm thick veneers. ^a^ and ^b^ indicate a *p-*value < 0.001 between the two concerned groups.

	MO Group (n = 10)	LT-A2 Group (n = 10)	MT-A2 Group (n = 10)	*p-*Value
Translucency Parameter (TP)	7.69 ± 1.39 ^a^	7.69 ± 0.91 ^b^	11.23 ± 0.9 ^a,b^	<0.0001

**Table 3:** Mean values (±SD) for the degree of conversion (DC) depending on the ceramic’s translucency. All the veneers have a 1 mm thickness.

Translucency	MO Group (n = 10)	LT-A2 Group (n = 10)	MT-A2 Group (n = 10)	*p-*Value
Degree of conversion (%)	59.75 ± 6.98	62.73 ± 4.63	65.71 ± 4.08	0.062

**Table 4:** Mean values (±SD) for the degree of conversion (DC) depending on the ceramic’s thickness. All the veneers have been designed with a MT-A2 opacity. ^a^, ^b^ and ^c^ indicate a *p*-value < 0.001 between the two concerned groups.

Thickness	0.6 mm (n = 8)	0.7 mm (n = 8)	0.8 mm (n = 8)	1 mm (n = 10)	*p-*Value
Degree of conversion (%)	87.46 ± 0.71 ^a^	87.44 ± 0.4 ^b^	86.46 ± 3.31 ^c^	65.71 ± 4.08 ^a,b,c^	<0.0001

## Data Availability

Data supporting reported results are accessible by simple request to the corresponding author.
